# Mitral Leaflet Flail as a Late Complication of Infective Endocarditis: A Case Report

**DOI:** 10.7759/cureus.25854

**Published:** 2022-06-11

**Authors:** Rafsan Ahmed, Amirhossein Moaddab, Suzette Graham-Hill

**Affiliations:** 1 Internal Medicine, State University of New York Downstate Health Sciences University, Brooklyn, USA; 2 Cardiology, State University of New York Downstate Health Sciences University, Brooklyn, USA; 3 Department of Cardiology, Kings County Hospital Center, Brooklyn, USA

**Keywords:** transesophageal echo, transthoracic echocardiogram, mitral regurgitation, miltral leaflet flail, infective endocarditis

## Abstract

Infective Endocarditis (IE) refers to an infection of the endocardial surface of the heart which leads to a wide array of complications, including heart failure, perivalvular abscess, metastatic infection, septic embolization, mycotic aneurysms, neurological and renal complications. Mitral leaflet flail (MLF), defined as a failure of leaflet coaptation with the rapid systolic movement of the involved leaflet into the left atrium, is a rare complication of IE which can lead to severe mitral regurgitation. Echocardiography plays a key role in making its diagnosis with transesophageal echocardiograms (TEE), providing greater sensitivity and specificity compared to transthoracic echocardiograms (TTE). MLF is often misdiagnosed, or diagnosis is delayed due to its presentation with non-specific cardiac symptoms. However, early diagnosis with echocardiography and prompt surgical correction leads to improved long-term survival. Here we have presented a case of a 71-year-old female with a past medical history of IE nine years ago who was referred to the cardiology clinic for one month of exertional dyspnea. TTE showed severe mitral regurgitation, and subsequent TEE confirmed flail mitral leaflet.

## Introduction

Mitral Leaflet Flail (MLF) is defined as failure of leaflet coaptation with the rapid systolic movement of the involved leaflet into the left atrium due to either ruptured chordae tendineae or papillary muscle. This may lead to acute, subacute, or chronic mitral regurgitation (MR) [1]. MLF may occur in patients of any age; however, it is more commonly seen in younger patients and males than females [2]. Mitral chordal rupture is the most common cause followed by myxomatous degeneration, infective endocarditis (IE), mitral annulus calcification, and papillary muscle rupture [2]. Papillary muscle rupture due to acute myocardial infarction (MI) is a rare cause [3]. Patients may present with sudden onset dyspnea, cough, chest pain, and a regurgitant murmur on physical exam. Echocardiography is the key modality for the diagnosis of MLF [4]. Here we have presented a case of a 71-year-old female with a past medical history of IE nine years ago who was referred to the cardiology clinic for one month of exertional dyspnea. TTE showed severe mitral regurgitation, and subsequent TEE confirmed MLF.

## Case presentation

**Figure 1:**
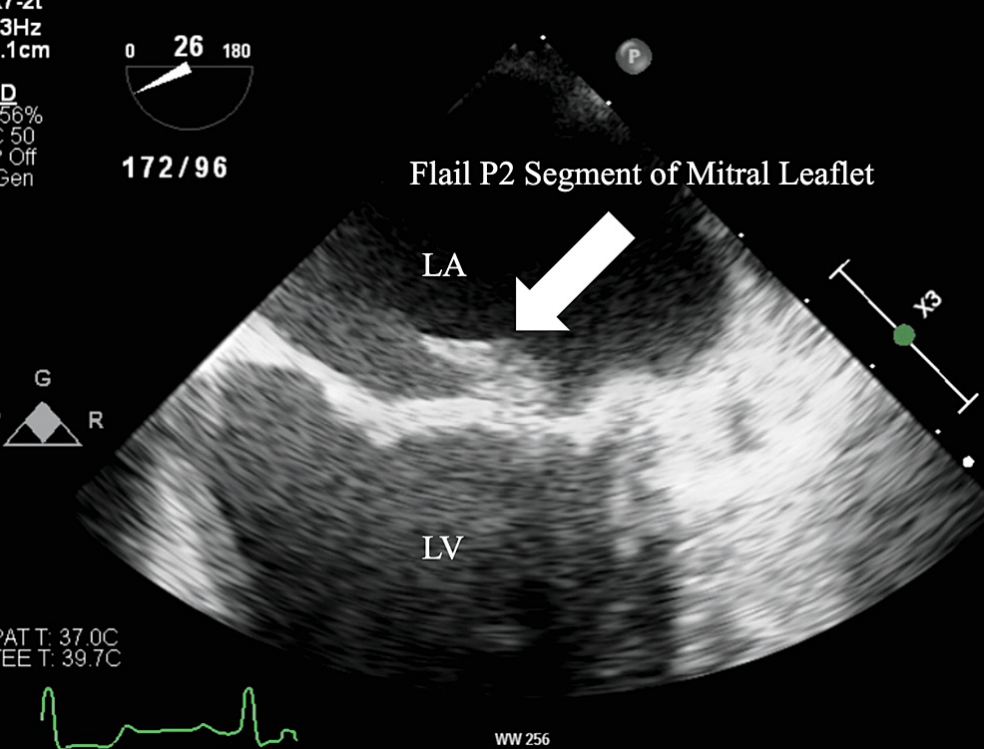
Mid-esophageal view of Transesophageal echocardiogram showing flail P2 portion of the mitral valve. LA: Left atrium, LV: Left ventricle

**Figure 2:**
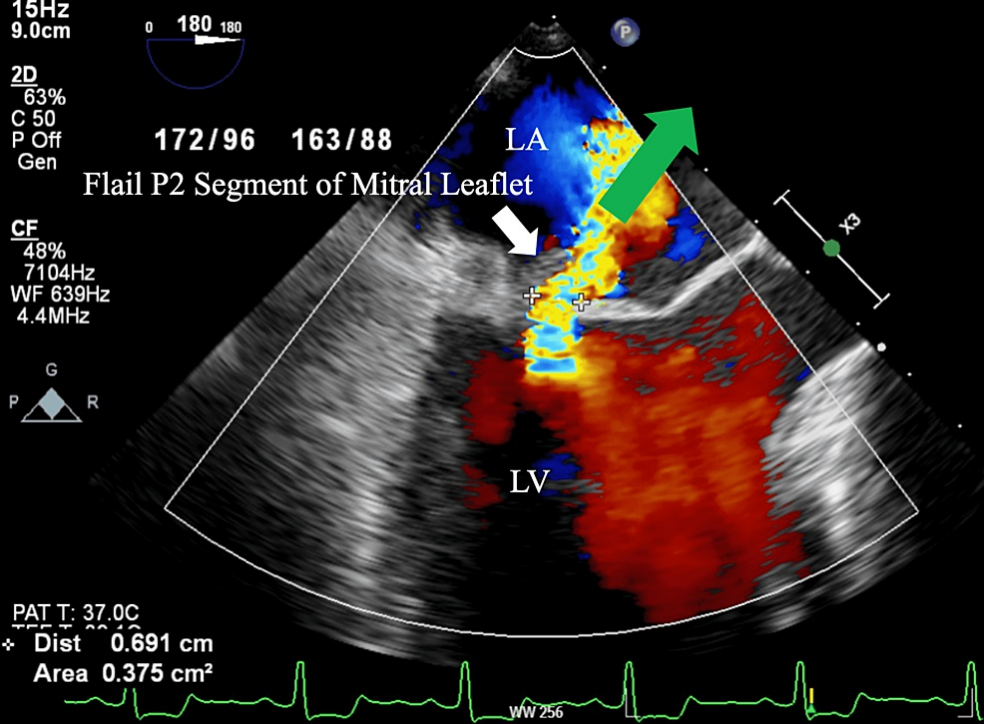
M-mode, mid-esophageal view of transesophageal echocardiogram showing flail mitral leaflet (white arrow) resulting in eccentric regurgitant flow (green arrow) from LV to LA. LA: Left atrium, LV: Left ventricle

A 71-year-old female with a past medical history of hypertension, hyperlipidemia, syphilis, glaucoma, and IE nine years before the presentation was referred to the cardiology clinic for one month of dyspnea on exertion. On further chart review, it was confirmed that she had echocardiographic evidence of mitral valve vegetation on the posterior leaflet nine years ago. A physical exam revealed a Grade II-III holosystolic murmur. A transthoracic echocardiogram (TTE) was done, which demonstrated the presence of preserved left ventricular ejection fraction (55-60%), moderately dilated left atrium (4.2cm), and severe mitral regurgitation. There was no evidence of myxomatous degeneration of the mitral valve. Subsequently, she underwent a transesophageal echocardiogram (TEE) which showed a flail portion of the posterior leaflet involving the medial scallop (P3) and middle scallop (P2) (Figure 1). TEE also showed an eccentric regurgitant flow from the left ventricle to the left atrium, which is characteristic of MLF (Figure 2). Considering the findings, surgical repair of the mitral valve was performed after coronary angiography showed non-obstructive coronary artery disease. During the surgical repair, MLF was confirmed with chordal rupture, and the flail portion of the P2 segment was excised and reconstructed. An annuloplasty and left atrial appendage clipping was performed as well. There were no complications throughout the procedure and on a follow-up to the clinic after recovery, the patient's symptoms had resolved.

## Discussion

IE refers to an infection of the endocardial surface of the heart, usually involving one or more valves or an intracardiac device [5]. It is associated with a broad array of complications, including heart failure, perivalvular abscess, metastatic infection, septic embolization, mycotic aneurysm, and neurological and renal complications [5]. In rare cases, bacterial endocarditis can destroy the aortic valve leaflets resulting in MLF [2,5]. Sudden onset dyspnea is a hallmark symptom of acute MLF and can be seen in approximately 50% of patients [4]. A physical exam is notable for a mitral regurgitant murmur in approximately 30% of patients [4]. During MR, the left atrium does not have enough time for compensatory dilation as a result, pulmonary edema develops [6]. Chest X-rays usually show bilateral pulmonary venous congestion, interstitial edema, and symmetrical bilateral pleural effusion [6]. Echocardiography is the key diagnostic modality in the diagnosis of MLF [7]. TEE shows greater sensitivity and specificity compared to TTE in visualizing MLF as it provides higher resolution imaging of posterior cardiac structures and Doppler color flow mapping [7]. Consistent with our case, MLF causes severe MR with the regurgitant blood entering the left atrium at an angle [2]. This is known as eccentric MR jet and is present in 78% of cases of MLF [2]. Early surgical correction is the treatment of choice and is associated with high overall survival at 10 years [5]. 

Here we have presented a rare case of a patient who presented with symptomatic mitral regurgitation because of MLF nine years after diagnosis of IE. To our knowledge, there are no reported cases of MLF that presented almost a decade after an incident of IE. Due to the wide array of symptoms caused by MLF, it is often misdiagnosed, or diagnosis is delayed leading to morbid outcomes. In this case, we highlight the severity of this valvular dysfunction, and we highlight the importance of echocardiography in its diagnosis when clinical suspicion is high. 

## Conclusions

MLF is a rare complication of IE which can cause acute, subacute, or chronic mitral regurgitation. MLF is often misdiagnosed, or diagnosis is delayed as symptoms can mimic a wide array of cardio-pulmonary diseases. Prompt diagnosis with echocardiography and surgical correction is key for long-term survival benefits.
